# Extirpation of Glandula Parotidea

**Published:** 1860-09

**Authors:** Gerhard Paoli

**Affiliations:** Chicago


### EXTIRPATION OF GLANDULA PAROTIDEA.

BY GERHARD PAOLI, M. D., CHICAGO.

Dr. Deville, of this city, called a public meeting, and through the city papers invited all medical men to listen to a lecture, to be delivered by himself, upon the Parotid Gland. The lecture seemed to be particularly intended to confute Dr. Brainard, who published, in his *Medical Journal* of December last, a very interesting original communication on this subject, wherein he plainly proved that he had several times removed the parotid gland. Dr. Deville seems to doubt the statements of our distinguished surgeon, and, indeed, doubts that the operation ever has been performed by any one, and quoted authors who question the possibility of the operation, without quoting a single one who ever has performed it. He stated that in all the medical museums in Paris and London he had never seen a single specimen of extirpated parotid gland. He doubted, indeed, whether there was any disease of that organ except hygroma. As to the anatomical facts his lecture was interesting, but in his surgical statements he came in conflict with experience; and I shall here, from an altogether scientific point of view, present a few facts in regard to this operation, and the diseases of this important organ. As far as I know, there is no special treatise upon the parotid gland, and I have, therefore, collected the following facts, which prove beyond a doubt, that the extirpation has been made many times, and furthermore, that the parotid is subject to disease as well as other glands. The particular character of the different kinds of tumors which appear in the region of the parotid are still very imperfectly understood; notwithstanding the researches of pathological anatomy, we are still very far from a clear understanding of the real seat and origin of them, of their diagnostic signs, their most rational treatment, or their more or less favorable prognosis, and it would be of great importance in practical medical science if we had more positive knowledge on these subjects.

I shall first, before attempting to prove that many distinguished surgeons have extirpated the parotid, briefly mention the diseases to which this organ is subject. To these belong carcinoma, osteoides, enchondroma, induration, hypertrophy, sialomo, hydroma, aneurism, lymphatic and many other tumors.

Carcinoma fibrosum produces a stone-like circumscribed swelling of less circumference than the gland, with uneven, knotted surface, is generally movable, grows slowly, and therefore may exist many years without causing the patient much trouble; sometimes it is very deeply seated and becomes spread out into the ear or throat.

Carcinoma medullare appears very seldom and produces larger tumors which grow more rapidly. On their first appearance they are like small, hard, uneven knots, which afterwards become softer, as if divided in small portions, and often present a spurious fluctuation. It is particularly this kind of carcinoma which causes the disease to take deeper root, where it can grow and distribute itself in different directions. In the case of a patient who died with a tumor of this description, Berard found that the internal jugular vein participated in the degeneration, and that the diseased mass reached into the vein. It can easily be seen how great the difficulty and danger would have been in this case to have attempted extirpation. A subdivision of this disease Rokitansky calls cystocarcinoma, whose nature does not differ materially from the other kinds. Prof. Larsen has removed a tumor of this kind from the parotid, and exhibited it in the Philatrien Medical Society.

*Induration.*—This form may be the consequence of chronic inflammation of the parotid, but in some cases it occurs without any such previous inflammation. Sometimes hard circumscribed tumors are found in the parotid, which, without causing any pain, or in any way particularly troubling the patient, continue unchanged many years. Such may be fibrous bodies, dermoides, enchondroma, or osteoides, and all appear to proceed from the fibrous elements of the gland. We are hardly likely ever to arrive at any certain signs by which they can be distinguished, till the anatomical knife, after operation, makes known to us their component parts. They are all considered of a mild nature, and can generally be easily removed without danger of revivification, with the exception of the osteoid, described by John Muller, which, according to Rokitansky, is nothing more than medullary sarcoma.

*Hypertrophy.*—There are very few instances of tumors of the parotid of this nature. Sabatier, who calls them the "*de la parotide*," has seen them twice; Tenon and Berard each once. They are often congenital affections, and depend upon the greater development of the separate portions of the gland so that they appear in a considerable compass, and form tumors more or less hanging. In some cases there was also a telangeiectasis or an increased vascularity of the glandular masses. In one of the cases mentioned by Sabatier, a man aged sixty had a large tumor on the side of the neck, with a constricted basis, so that it was easily moved in all directions, its insertion reachiug from the zygomatic arch and the ear, which was projected forcibly forward over the masseter muscle. It had existed fourteen years and grew very solwly in the beginning, but afterwards more rapidly. He removed it successfully with a ligature, and compared the inner structure to that of chronic hypertrophy of the tonsils. Tenon saw a case of a child of one year, where the tumor was about the size of a man's fist. On the death of the child, which occurred soon after, the tumor was fouud to have been caused by a derangement of the parotid, which also was full of strangely developed arteries which proceeded from the external carotid. Berard saw a child two or three years of age, who had a similar tumor, having its seat in the region of the parotid. It was oblong, tolerably hard, of a bluish color when the child cried or exerted itself. The compass could be diminished by compression. All the physicians who saw it considered it a telangeiectasis. Berard was just about treating it as such, when the child died of another disease. It was then found that the tumor consisted of a hypertrophied parotid whose texture was unchanged, but granulations were interwoven with a quantity of strangely developed veins.

*Sialomo.*—These tumors, which are also described under the name of abscesses, or tumores salivales, consist of a collection of thick saliva, contained in a closed sac, in the parotid, but instances of these are so rare, that in the annals of medical science very few are recorded; they are the same for the parotid, as ranula for the sublingual gland. These tumors sometimes contain sand, or small calculi salivals; they differ in size from a nut to a goose egg, and even larger. Felix Plater describes such an one Lib. 3, p. 689. Keemer describes a similar tumor. (*Journal fur Chirurgie Augenheikunde* of Graeffe and Walter.

*Hygroma—Rokitansky.*—These also appear very seldom, and are distinguishable from sialomo by their not containing saliva, but a thin, divers-colored fluid, clear as water, which is contained in a sac. Henry* observed such a tumor in a child four years of age, which had suffered therefrom from its earliest years. It developed slowly without pain, together with a feeling of fluctuation; at the first attempt to remove it, six ounces of a clear fluid flowed out. Valker and Wutzer have described a similar case.

*Aneurism.*—Tumors of this kind proceed from lesions of the external carotid, or its branches, or of neighboring arteries, as the vertebral; they may be of considerable circumference, and their diagnosis is sometise attended with great difficulty, as they do not always pulsate, and under the form of a false aneurism may produce tolerably hard tumors contained in the parotid, and surrounded by its aponeuroses. They appear oftenest after lesion of the arteries consequent upon shot wounds, etc. Berard saw them in the upper part of the region around the parotis after arteriotomy. A remarkable tumor of this kind is described by Jovil.† The patient, in consequence of a shot wound received in a duel, had a lesion of the external carotid and internal jugular vein, whereby these blood-vessels were brought into internal communication, but the artery also opened in an aneurismatic sac of the size of a pigeon egg, which formed a considerably large tumor in the region of the parotid, so that there existed at the same time both varix aneurisma and aneurisma varicosum. Besides, Romagna,‡ of Naples, has communicated a not less remarkable case, where a tumor in the parotid gland proceeded from an aneurism of the vertebral artery caused by a punctured wound under the ear. The projection, a false aneurism, formed a considerable sized tumor, which reached from the ear to the angle maxilla inferior, and from the edge of the sterno cleido mastoideus to the edge of the maxilla inferior.

The principal tumors in the parotid, whether they proceed from the degeneration of the gland itself, or of the lymphatic glands, have generally several similar qualities, such as growing slowly in the beginning, or remaining stationary a long

---

* Bulletin de l'Academie.

† Gazette Medicale, p. 457: 1840.

‡ Archives General de Medicin, 2d series, 5th volume, page 138.

time; and as they are frequently wedged in between the processus mastoideus and maxilla inferior, lift the ear more or less upward, and thereby the lobulus of the ear sometimes almost disappears. When the tumors have reached a considerable size, they sometimes impede the motion of the maxilla, press together the ear channel, and affect the hearing, as well as compress the large blood-vessels, thereby causing a feeling of giddiness in the head. They soon almost imperceptibly make their appearance in the form of little hard moveable knots in the region of the parotid. Preeger thus describes a case where a woman thirty-three years of age suddenly, at night, felt an acute pain in the cheek, which reached down to her neck, and was so severe that she could not raise her head without assistance; sometime after, she observed a little knot under her ear, which was tender to the touch, and then, after a lapse of ten years, it had grown to an immense size, which not only covered her cheek, but reached down to her breast, parallel with the pharynx, which it pressed aside. Burns observed a similar tumor.

*Extirpation.*—This operation, the most customary and certain relief for the greater part of the tumors which occur in this region, was the subject of much discussion and strife among surgeons at the close of the past and beginning of the present century; but we now, finally—partly by the aid of discoveries in pathological anatomy, and partly from the teachings of experience—seem to have arrived at some definite results. A number of older sugeons, such as Heister, Schaarshmidt, Schultet, Acrel, Siebold the elder, Werdier, Sarengeot, Roonhuysen, and several others, have declared that they have extirpated the whole parotid, and give a number of observations, which conclusively prove the truth of their statements.

Notwithstanding all this, Burns and Ritcher, and afterwards Boyer, attempted to prove that these surgeons, when they believed they had extirpated the parotid, had deceived themselves. They attempted first to show that the tumors described by them were not generally seated in the parotid, but in the adjacent lymphatic glands, which pressed the parotid down in the cavity and atrophied it, and after their removal it seemed as if the whole parotid had been removed. Richter even went so far in condemnation of the operation, that he did not consider a surgeon warranted in performing such a hazardous operation. Still, many of the older surgeons have proven by their own observations, that some at least of the tumors in the parotid have been removed with complete success, and without danger of lesion of any consequence, even in many cases without the slightest difficulty, and that it can be done without removing the whole of the parotid, in which they have developed themselves.

So the possibility of total extirpation being performed, has been established beyond a doubt, by many surgeons whose names occupy a prominent place in the annals of medicine, and in this connection, I will here mention some of these distinguished surgeons, viz: Beclard in 1823, Randolph, Lisfranc, Gensoul, Hendricks, Ollenrath, Kleins, Ohle, Walter, Chelius, Prieger, Berendts, Weinhold, Beelard, McClellan, Dieffenbach, Heister, Wallman, in 1828, in Vienna; Dupuytren, in 1829, in Paris; Bendz, in Copenhagen, in 1838; Verdier, Mott, Geyrond, Boyer, Stedman, Lacoste, Larrey, Nægle, in 1830; Brainard, in Chicago, and many others.

These all prove that the parotid can be removed, even when the glands are degenerated, and in fact they recommend the operation as being absolutely necessary. That it can be done is much the more indisputably proven by careful examination immediately after the operation, when the cavity of the parotid region, blood-vessels, nerves, and parts bordering on the cavity inside, may be seen without any remnants of the glands, partly also by consequent perfect paralysis of the face, and partly by post-mortem examination. At the same time experience teaches that degeneration of the parotid can thus take place when prolongations of the diseased mass have forced themselves in the deepest parts, for instance; to the bassilary process, or atlas, then the complete extirpation of the gland becomes impossible, still science may accomplish much under very difficult circumstances. Hendricks has shown, in a case where the tumor was very large, and unusually firm, and sent prolongations into the deepest part, among blood-vessels, nerves, muscles, etc., that one of these prolongations hung fast to the stem of the nervus vagus, and had to be separated by very careful dissection, the pharynx was seen entirely bared, contracting itself together under the constant desire to swallow, which distressed the patient. Under this troublesome operation, which lasted an hour and a quarter, several arteries were tied, but the lesion of the carotid was avoided.*

These questions may now be considered conclusively settled:

1. That tumors lying in the parotid have been successfully removed without the necessity of removing the whole gland.

---

* Braamberg Dis de exterpatione gland parotis, et sub maxillaris, 1829.

2. That the whole parotid can be extirpated when circumstances require it, either when the whole gland is degenerated, or when a tumor, on account of its situation, cannot be removed without taking the parotid also. Practical talent and experience are what are most to be relied upon in such cases, but we may consider it a tolerably well established rule, that in every tumor which we have not been able to disperse, or where dispersion is not advisable, there is an indication for extirpation.

Neither the size of the tumor, nor its ulceration, nor the age of the patient, are any contraindications to the operation, for many surgeons have extirpated the largest tumors with complete success. Some have thought best not to operate on scrofulous subjects, or lymphatic tumors, because they consider scrofula a constitutional disease and that the operation would be of no use. In the meantime, Sidellet has undertaken a number of operations of this kind among soldiers, as also Velpeau and Warren, and experience seems to have taught them to consider these tumors in patients who have arrived at a certain age, more as local affections than constitutional diseases. Upon these grounds they removed others with equal success from other parts of the body, for instance, the groin, axilla, neck, etc. Still the operation must be considered as contraindicated when the tumor is very deep-seated, or there are suspicions that it reaches very deep, and has a considerable prolongation, for instance, reaches forward in the fauces behind the tonsils.

That they are carcinomatous, ought not to deter us from attempting their removal, as experience has shown by many observations that these tumors have been successfully removed without danger of their returning; melanosis is an exception to this, and must be considered as a *noli me tangere*.

Before we proceed to extirpation of deep seated tumors, which often expose the parotid, the question should be well considered, whether we can insure ourselves against hemorrhage by ligature of the common carotid beforehand. This proposition was first made and executed by the English physician, Goodland* while extirpating the whole parotid, and several have followed his example. It is not easy to say beforehand whether the tumor is formed by degeneration of the whole or a part of the parotid, which of course would have more or less effect upon the hemorrhage, as experience shows us that many tumors in the lymphatic glands can be removed

---

* Medical and Surgical Transactions, vol. i: p. 12, 1819.

without the least danger of producing lesion of the carotid. Also, the situation of the artery is variable, sometimes perforating the gland's deepest part, sometimes lying in a cavity at its point, or the foremost edge, and as one position occurs quite as frequently as the other, it follows that even in the extirpation of the whole parotid, it is not always necessary to cut the external carotid, which for instance, Smidt, Lisfranc, and Hendricks avoided, and McClellan was even so fortunate as to loosen the whole artery out of the diseased mass in which it lay, and extirpated the whole gland without the necessity of tying it; furthermore, several surgeous state, that tying the common carotid has, in several cases not only proved to be a difficult and dangerous operation, but its consequences have been injurious to some of the vital organs, particularly the brain, lungs, larynx; and the internal carotid, can, in this operation, almost always be avoided. Velpeau very justly observed, that this preliminary ligature ought, under all circumstances, to be confined to the external carotid, and besides assures us that he never has found it necessary to undertake any such ligature, although he has constantly performed this kind of operation, and in doing so, often reached in to the very depths of the parotid cavity. For these reasons it is not only unnecessary to place a ligature upon the external carotid, but in many cases it would be connected with great difficulties, because the lower end of the tumor generally covers the artery. On this account it is sometimes difficult enough to place a ligature upon the common carotid, and we see, for instance, that Dr. Stedman occupied fifty-five minutes in doing it. In attempting to pass such a ligature, he happened to open the jugular vein, and thereby nearly lost his patient through the entrance of air into the vein.

Berard also considered this preliminary ligature quite unnecessary except in case of a large encephaloide, which tumors are sometimes very frail, and if torn under the operation might produce dangerous parenechymatose hemorrhage. Instead of a preliminary ligature a reserve ligature (ligature d'attende) under the common carotid, has also been recommended as advisable under certain circumstances. Beelard, Roux, Lisfranc and Gensoul have made use of it. Langenbeck and Braamberg compress the artery during the operation with the finger.

All now seem agreed that these precautionary measures are unnecessary, and depend upon reliable assistants who, during the operation, can compress the primitive artery.

It is generally acknowledged that definite surgical rules are sometimes very difficult to establish. In such cases talent and skill only can guide the surgeon, and he is often obliged to create his own methods, and difficult as the operation seems, and to many it may appear an impossibility, still, no one who is acquainted with the surgical annals of different countries can doubt the records of men whose only object has been to relieve suffering, and leave truthfully to posterity their experience.—*Louisville Medical News.*

